# The mystery of massive mitochondrial complexes: the apicomplexan respiratory chain

**DOI:** 10.1016/j.pt.2022.09.008

**Published:** 2022-10-24

**Authors:** Andrew E. Maclean, Jenni A. Hayward, Diego Huet, Giel G. van Dooren, Lilach Sheiner

**Affiliations:** 1 Wellcome Centre for Integrative Parasitology, University of Glasgow, Glasgow, UK; 2 Research School of Biology, Australian National University, Canberra, Australia; 3 Center for Tropical & Emerging Diseases, University of Georgia, Athens, GA, USA; 4 Department of Pharmaceutical and Biomedical Sciences, University of Georgia, Athens, GA, USA

## Abstract

The mitochondrial respiratory chain is an essential pathway in most studied eukaryotes due to its roles in respiration and other pathways that depend on mitochondrial membrane potential. Apicomplexans are unicellular eukaryotes whose members have an impact on global health. The respiratory chain is a drug target for some members of this group, notably the malaria-causing *Plasmodium* spp. This has motivated studies of the respiratory chain in apicomplexan parasites, primarily *Toxoplasma gondii* and *Plasmodium* spp. for which experimental tools are most advanced. Studies of the respiratory complexes in these organisms revealed numerous novel features, including expansion of complex size. The divergence of apicomplexan mitochondria from commonly studied models highlights the diversity of mitochondrial form and function across eukaryotic life.

## The mitochondrial respiratory chain of apicomplexan parasites

**Figure 1.**
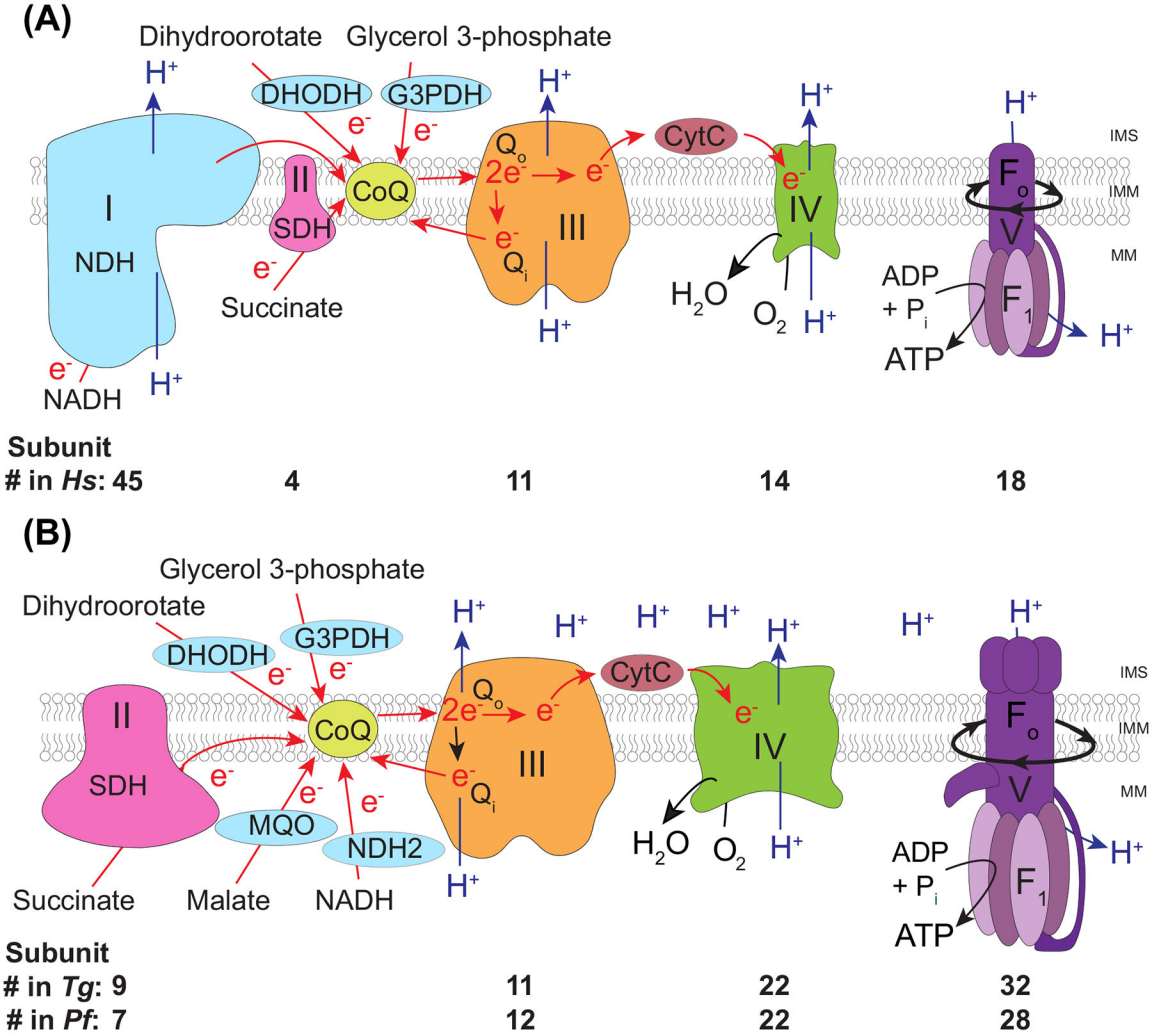
Comparison of the respiratory chain of humans (A) and apicomplexans (B). Dehydrogenases and oxidoreductases oxidise a range of mitochondrial substrates and donate electrons to coenzyme Q (CoA). These enzymes include the multisubunit Complex I in humans (NADH dehydrogenase), single-subunit NADH dehydrogenases in apicomplexans (NDH2), Complex II (succinate dehydrogenase (SDH), dihydroorotate dehydrogenase (DHODH), and glycerol 3-phosphate dehydrogenase (G3PDH). Reduced CoQ docks at the Q_o_ site of Complex III and releases two electrons. One electron is donated to cytochrome *c* (CytC) while the other is donated back to CoQ at the Q_i_ site during the so-called Q-cycle. CytC transports the electron to Complex IV, which passes the electron to the final acceptor, oxygen (O_2_), to form water (H_2_O). Protons (H^+^, blue arrows) are pumped through Complexes I, III, and IV from the mitochondrial matrix (MM) into the intermembrane space (IMS) to form a proton-motive force across the inner mitochondrial membrane (IMM). F_o_-F_1_ ATP synthase (Complex V) exploits this proton gradient to power rotation of the F_0_ domain as a proton re-enters the MM, and couples this movement to the activity of the catalytic F_1_ domain to synthesise ATP. Complexes II, III, IV, and F_o_-F_1_ ATP synthase of apicomplexans are larger and contain numerous additional subunits, compared to the equivalent complexes in humans (see text and beneath each complex for details).

The **mitochondrial electron transport chain** (**mETC**, see Glossary; Figure 1) and **F_o_-F_1_ ATP synthase**, often collectively referred to as the respiratory chain, are a series of protein complexes in the inner **mitochondrial** membrane (IMM), that plays central roles in **oxidative phosphorylation** (**OXPHOS)**. The proton gradient generated by the mETC is also important for other cellular processes such as mitochondrial protein import and pyrimidine biosynthesis. In OXPHOS, electrons are harvested from metabolic pathways involving carbohydrates, fats, and amino acids, and are fed into the mETC. As electrons are transferred to the final electron acceptor, oxygen, protons are translocated across the IMM. F_o_-F_1_ ATP synthase then utilises the resulting proton gradient for the synthesis of ATP. OXPHOS is one of life’s most important reactions, and thus much research effort over the past decades focused on understanding the respiratory chain in detail, with key advances leading to several Nobel prizes, such as the 1978 prize in chemistry for Peter Mitchell. This was awarded for his work on the chemiosmotic theory allowing insight into the process of OXPHOS and is considered one of the great scientific achievements of the last century. Historically, most of the attention focused on the **Opisthokonta** clade of eukaryotes, which includes mammals and yeast. For this reason, the protein content, composition, and structure–function features of the respiratory complexes in those organisms are understood in great detail and dominate higher-education textbooks. However, this biased focus is changing to include a broader and more diverse repertoire of model systems. Recent studies, in particular structural studies, in a diverse range of unicellular eukaryotes, for example *Tetrahymena* and *Paramecium* (ciliates) [1-5], *Euglena* and *Trypanosoma* (euglenozoan) [6,7], *Polytomella* (algae) [8], and *Toxoplasma* (**Apicomplexa**) [9], underlie the value in an expanded pool of mitochondrial models. Here, we review the findings from Apicomplexa, and other **Myzozoa**, and highlight their differences to the canonical ‘textbook’ system.

Myzozoans are a group of unicellular eukaryotes that include the dinoflagellates, aquatic plankton whose bloom may have an impact on marine and freshwater safety; perkinsozoans, parasites of marine animals whose infection has economic impact, for example on the oyster industry; chromerida, photosynthetic symbionts of corals, with impact on marine ecology; and apicomplexans, a phylum of human and animal parasites causing severe diseases such as malaria, caused by ***Plasmodium*** spp., and toxoplasmosis, caused by ***Toxoplasma gondii***.

The apicomplexan respiratory chain has been studied for many years, largely due to being the target for drugs, such as atovaquone [10]. However, challenges in working with parasites made it prohibitively hard to perform the large biochemical and structural studies routinely carried out to study respiratory chain properties in other model systems. Advances in biochemical, proteomics and microscopy methods led to renewed efforts in recent years. It now has become apparent that the apicomplexan respiratory complexes show remarkable differences to the opisthokont complexes, especially in size and composition. Perhaps contrary to an instinctive expectation, these protist respiratory complexes often contain more subunits than their human and other metazoan parallels. Here we review the composition of the apicomplexan respiratory chain complexes, discussing the differences that exist between the apicomplexan pathway and that of metazoans, and why these differences might have arisen.

## Dehydrogenases: multiple entry points into the mETC

Many eukaryotes, including well-studied mammalian and plant models, have a multisubunit proton-pumping Complex I (type I NADH dehydrogenase, NADH:ubiquinone oxidoreductase), typically comprising between 40 and 50 protein subunits [11-13], but with some larger examples, including from the ciliate *Tetrahymena thermophila*, which is in the sister group to myzozoans, which has 68 or 69 subunits [2,3]. Complex I oxidises NADH and transfers electrons to Coenzyme Q (also known as ubiquinone) (Figure 1). This process is coupled with the pumping of protons across the IMM. In mammals, this complex is responsible for an estimated 40% of the proton-pumping capacity of the mETC [14]. Apicomplexans, and indeed all myzozoans, lack this multisubunit complex: their genomes lack Complex I-encoding genes [15,16] and they are insensitive to the Complex I inhibitor rotenone [17-19]. Instead, matrix-facing single-subunit type II NADH dehydrogenases (NDH2) fulfil the role of oxidising NADH and transferring electrons to Coenzyme Q [20-22]. However, unlike Complex I, NDH2 does not contribute to proton pumping. No known complex in myzozoans compensates for the proton-pumping ability of Complex I, and the effect of this potential loss on the proton-pumping capacity of the myzozoan mETC remains a question for future study. Humans do not have NDH2, and for this reason apicomplexan NDH2 has been the focus of drug-discovery studies [23-25]. The events that led to the loss of Complex I in the myzozoan lineage are unknown, but one hypothesis points to a benefit in the reduction of superoxide generation [26]. Loss of Complex I is not unique to myzozoans [27-29] and it remains to be determined whether the same, or different, selective pressures led to the loss in each lineage.

Electrons can also enter the mETC via other single-subunit dehydrogenases, including dihydroorotate dehydrogenase (DHODH), FAD-dependent glycerol 3-phosphate dehydrogenase (G3PDH), and malate:quinone oxidoreductase (MQO) (Figure 1). MQO is conserved across myzozoans [15,30] but is absent from the mammalian hosts, again leading to interest in MQO as a drug target. Both G3PDH and DHODH, which are thought to localise to the outer face of the IMM, are also found in mammals. Nevertheless, both are candidate drug targets. Apicomplexan DHODH has a well-documented essential role in pyrimidine biosynthesis [31-33] and is the target of drugs undergoing clinical trials [34-36]. Apicomplexan G3PDH’s drug target candidacy is due to amino acid substitutions in critical domains compared to the mammalian orthologue [37]. All four of these dehydrogenases are expressed and localise to the mitochondrion in the tachyzoite life stage of *T. gondii* and *Plasmodium* blood stages [38-42]. They are therefore likely to function in the disease-causing stages of this these parasites.

## Complex II: multiplicity of subunits, rule or exception?

Complex II, the second multisubunit complex of the opisthokont mETC, is another major entry point of electrons into the mETC (Figure 1). Complex II is a succinate dehydrogenase (SDH) enzyme embedded in the IMM, facing the matrix. Uniquely among mETC complexes, it is part of the tricarboxylic acid (TCA) cycle. Complex II oxidises succinate to fumarate in the mitochondrial matrix, whilst transferring electrons, via a flavin adenine dinucleotide (FAD) cofactor and three 2Fe-2S clusters, to reduce Coenzyme Q, which then shuttles electrons onward to Complex III [43,44](Figure 1). The composition and structure of Complex II is well characterised in a number of fungal and mammalian models. The opisthokont complex consists of four subunits: SDHA and SDHB make up the soluble matrix-facing domain, and SDHC and SDHD are integral membrane proteins which attach the complex to the inner membrane. SDHA contains the succinate binding site and FAD cofactor, while SDHB contains three 2Fe-2S clusters. SDHC and SDHD anchor the complex in the IMM and contain a heme *b* prosthetic group and a Coenzyme Q binding site[43,44]. Unlike for Complex I, III, and IV, electron transfer is not coupled to proton translocation, and so Complex II does not contribute directly to the proton-motive force.

Until recently, little was known about the composition and function of Complex II in myzozoans. The two well-conserved matrix subunits, SDHA and SDHB, were readily identifiable by homology searches in most apicomplexans [15,45]. However, some apicomplexans, including some *Cryptosporidium* species, have lost Complex II [46,47]. These groups have highly reduced mitochondria, termed mitosomes, and mETCs, and so the loss of Complex II is consistent with an overall decrease in mitochondrial function and respiratory capacity. It was presumed that homologues of SDHC and SDHD had diverged in sequence to such an extent that they were not readily identifiable by common bioinformatic tools. Two subunits in *Plasmodium* were suggested as putative membrane-anchoring subunits based on the presence of sequence features suggestive of heme and Coenzyme Q binding [48,49]. However, it was not until several proteomic studies were carried out that the identities of the potential membrane-anchoring subunits were revealed. The first suggestion – that apicomplexans have a Complex II that is different from the opisthokonts – came from a study in *Eimeria tenella*, revealing a 745 kDa Complex II [50]. This is much larger than the ~130 kDa seen in mammals [51] and suggested that Complex II in apicomplexans possibly contained additional subunits. Two complexome profiling proteomic studies in *T. gondii* [38] and *Plasmodium falciparum* [41] identified complexes that migrated at 500 and 530 kDa, respectively. Interestingly, it appeared that they both contained many more subunits than the four-subunit complex: *T. gondii* Complex II was found to putatively contain nine subunits and *P. falciparum* seven subunits. These extra subunits still did not fully account for the observed increased mass, suggesting that the apicomplexan Complex II may migrate as a trimer or multimer. Neither of the *Plasmodium* proteins identified by previous sequence analysis [48,49] were found to comigrate with other Complex II subunits, suggesting that they are unlikely to be part of this complex. The absence of clear SDHC and SDHD homologues, and the presence of more than four subunits, occurs in other eukaryotes: 12 subunits in *Trypanosoma cruzi* [52], 8 in the plant species *Arabidopsis thaliana* [53,54], and 15 in the ciliate *T. thermophila* [3]. In plants, it was suggested that the multiple new subunits play a role in anchoring the complex in the membrane, thus replacing SDHC and SDHD. The same may well be happening in apicomplexans. One support for this possibility is the finding that one of the newly identified *P. falciparum* subunits contains a ‘DY’ motif [41] that is commonly seen in SDHC and has a role in Coenzyme Q binding [55]. The *T. gondii* homologue subunit also contains this DY motif, providing further support to this hypothesis, which future functional studies would address. The exact roles of these newly identified subunits will require further study.

## Complex III: an important drug target

Complex III (also called the Coenzyme Q:cytochrome *c* oxidoreductase or cytochrome *bc*_1_ complex) couples the net transfer of electrons from reduced Coenzyme Q to cytochrome *c* with the net movement of protons across the IMM [56] (Figure 1). The catalytic core of Complex III is formed by three proteins: the mitochondrial genome-encoded cytochrome b, which spans the mitochondrial membrane and forms the so-called Q_o_ and Q_i_ sites at which Coenzyme Q molecules bind; and the nuclear genome-encoded cytochrome *c*_1_ and Rieske subunits. The latter two, together, function in transferring electrons from Coenzyme Q at the Q_o_ site to cytochrome *c*. Coenzyme Q oxidation at the Q_o_ site releases two electrons. One electron is passed via the two heme prosthetic groups of cytochrome *b* to oxidised Coenzyme Q at the Q_i_ site in a process termed the Q-cycle [57]. The other electron is passed via the iron-sulfur cluster of the Rieske protein and the heme group of cytochrome *c*_1_ to cytochrome *c*. In addition to the three catalytic subunits, a further seven noncatalytic or ‘supernumerary’ subunits are found in yeast [58] and eight in mammals [59,60], which are proposed to stabilise the complex and/or contribute to the interactions with Complex IV within a Complex III–Complex IV **supercomplex** formation [51]. Complex III forms a dimer in all organisms studied to date.

**Figure 2.**
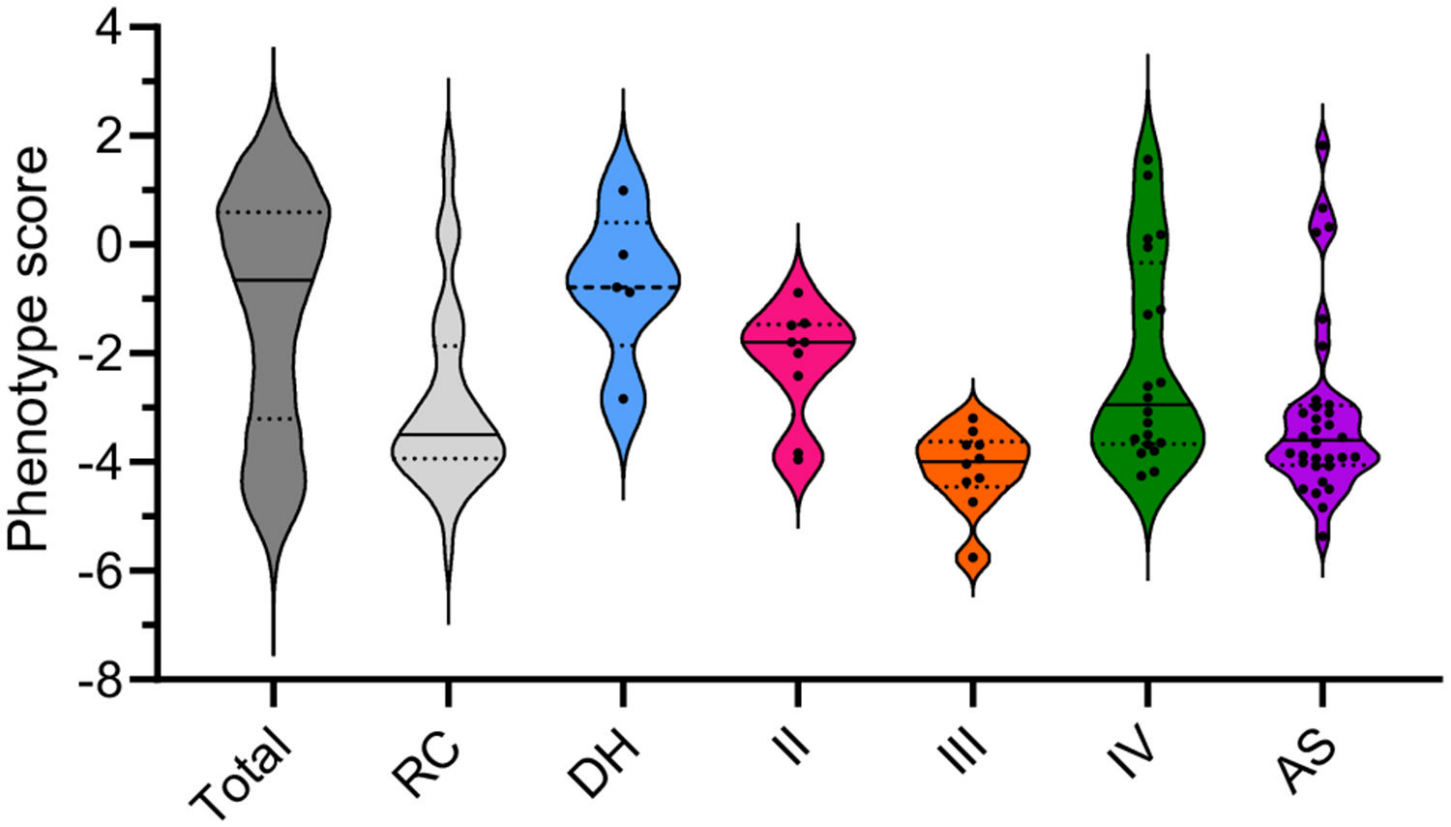
Predicted importance of the mitochondrial respiratory chain for *Toxoplasma gondii* survival. Phenotype scores were obtained from a genome-wide clustered regularly interspaced short palindromic repeats (CRISPR) screen of *T. gondii* parasites. The lower the phenotype score, the more important a gene is predicted to be for parasite proliferation. The overall distribution of phenotype scores are shown for all *T. gondii* genes (Total: dark grey) and for genes encoding respiratory Complex II–V subunits (RC: light grey). The distribution of phenotype scores for the dehydrogenases (DH blue), Complex II (pink), Complex III (orange), Complex IV (green), and F_o_-F_1_ ATP synthase (AS purple) subunits are then displayed individually, with each subunit represented by a dot. The median is indicated by a unbroken black line, while the upper and lower quartiles are indicated by broken lines. Genes that are important for parasite proliferation typically have phenotype scores of <−2.

**Figure 3.**
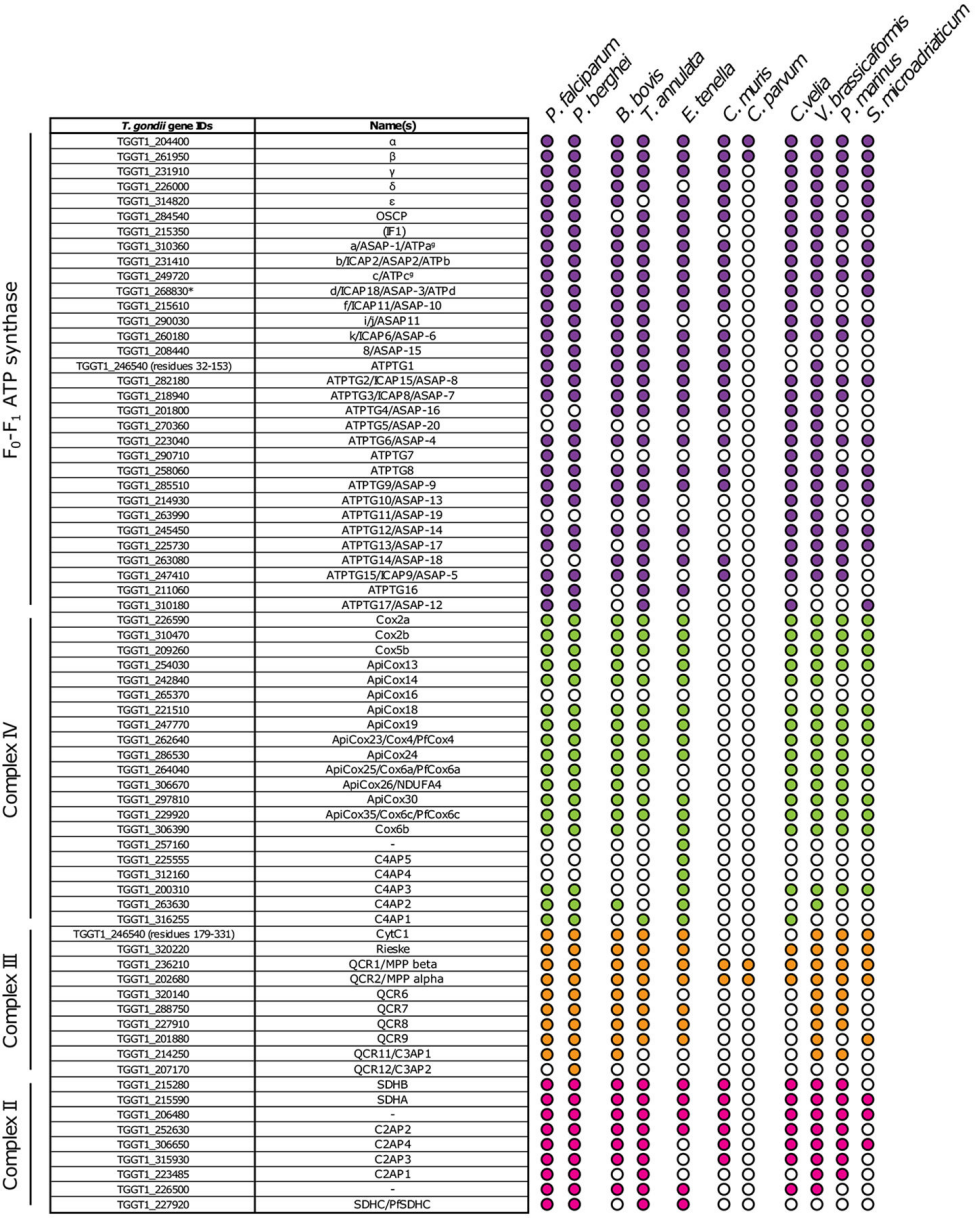
Mitochondrial respiratory chain subunit composition across different myzozoan species. Diagram showing the gene IDs and the names of all the nuclear encoded subunits found in Complex II (pink), Complex III (orange), Complex IV (green), and F_o_-F_1_ ATP synthase (purple) of *Toxoplasma gondii*. Subunit names from different publications and from *Plasmodium falciparum* are also included. On the right, their conservation in other ten myzozoan species (*Plasmodium berghei, Babesia bovis, Theileria annulata, Eimeria tenella, Cyryptosporidium muris, Cryptosporidium parvum, Chromera velia, Vitrella brassicaformis, Perkinsus marinus*, and *Symbiodinium microadriaticum*) is represented with a filled circle and their absence with an empty one. Homology to the subunits was performed through BLAST searches. Evidence for the complexes is supported at a proteomic level in all organisms, and only the structure of the *T. gondii* F_o_-F_1_ ATP synthase has been resolved to date.

The importance of Complex III as a drug target in apicomplexans has long been appreciated, with the Q_o_ site inhibitor atovaquone used to treat toxoplasmosis and malaria [61,62]. While Q_o_ site mutations in cytochrome *b* that confer atovaquone resistance have been studied extensively [63-65], the precise mode of atovaquone binding has not been addressed directly. Recently, two independent studies elucidated the composition of *T. gondii* Complex III through proteomic approaches [38,66]. Both studies identified 11 *T. gondii* Complex III subunits, including 4 novel or divergent proteins compared to the equivalent opisthokont complex. All 11 subunits are predicted to be important for parasite proliferation [67] (Figure 2). Two appeared to be restricted to apicomplexans (*Tg*QCR12) or myzozoans (*Tg*QCR11), and two had some similarity to Complex III subunits from other organisms (*Tg*QCR8 and *Tg*QCR9) [38,66] (Figure 3). All four divergent subunits were shown to be important for parasite proliferation and for the integrity of Complex III, with *Tg*QCR11 demonstrated to be essential for Complex III activity [38,66]. The composition of *P. falciparum* Complex III was also elucidated by the recent complexome profiling [41]. Homologues of all 11 *T. gondii* subunits were identified, including homologues for *Tg*QCR11 (C3AP1) and *Tg*QCR12 (C3AP2), plus an additional subunit (C3AP3) not identified in *T. gondii* Complex III [41]. The molecular mass of Complex III in *T. gondii* was estimated to be ~670 kDa [38,66], while the *P. falciparum* complex was slightly larger at ~730 kDa [41], both approximating the mass of dimeric Complex III from other organisms [68].

## Complex IV: an expanded terminal oxidase complex

Complex IV (also called cytochrome *c* oxidase) couples the transfer of electrons from cytochrome *c* to oxygen with the translocation of protons from the mitochondrial matrix into the intermembrane space [69,70] (Figure 1). The core of the complex is composed of three hydrophobic proteins: Cox1, Cox2, and Cox3. In mammals and yeast, all three are encoded on the mitochondrial genome. The catalytic subunits Cox1 and Cox2 contain heme and copper cofactors that transfer electrons from cytochrome *c* to oxygen [71]. While Cox3 does not participate in electron transport directly, it is thought to have regulatory and stabilising roles [72,73]. A further 11 noncatalytic subunits in mammals [74], and nine or ten in yeast [75], are encoded on the nuclear genome and assemble around the three core subunits in an intricate biogenesis process [70]. The noncatalytic subunits function to stabilise the complex, regulate complex activity, and mediate the formation of supercomplexes [51,76].

Complex IV composition in apicomplexans differs considerably from other organisms. Early studies found that Cox2 is split into two separate proteins (Cox2a and Cox2b) that are encoded in the nuclear genome of apicomplexans [77,78], reminiscent of what is found in some green algae [79]. A proteomic analysis of Complex IV from *T. gondii* identified five conserved subunits and a further 11 novel or divergent subunits that were termed ‘ApiCox’ proteins [40]. All of these proposed Complex IV subunits – with the exception of *Tg*ApiCox16 – are predicted to be important for *T. gondii* proliferation [67] (Figure 2), and *Tg*ApiCox25, a divergent homologue of the mammalian Cox6a protein, was shown to be critical for *T. gondii* mETC activity. The *T. gondii* complexome study identified the same 16 proteins, providing validation to the previous study, and an additional six candidate subunits [38]. Interestingly, these additional six subunits are predicted to be dispensable for *T. gondii* proliferation in culture [67] (Figure 2) but their importance for growth *in vivo* or under nutrient deprivation [80] is yet to be assessed. The *P. falciparum* complexome study identified homologues to 21 of the collective 22 *T. gondii* putative Complex IV subunits [41], along with an additional subunit not identified in *T. gondii* (PF3D7_0928000) that is likely a homologue of the mammalian Cox6b protein. Most of the novel ‘ApiCox’ Complex IV subunits are restricted to apicomplexans and other myzozoans (Figure 3), an exception being *Tg*ApiCox13 which has homology to the mammalian iron-sulfur domain-containing CISD3 protein [40,81], a protein that is not a component of Complex IV in mammals. The molecular mass of *T. gondii* Complex IV is estimated to be between 460 kDa [38] and 600 kDa [40,82], while that of *P. falciparum* is ~570 kDa [41], which is considerably larger than the equivalent complex from mammals and yeast (~200 kDa) [51,83].

Why Complex IV has expanded in apicomplexans remains unknown. It is likely that some subunits mediate the complex integrity or assembly. In support of this hypothesis, knockdown of *Tg*ApiCox25 led to the breakdown of Complex IV into a smaller, nonfunctional complex containing *Tg*Cox2a [40,66]. Additional functional and structural work is required to further examine this hypothesis.

## F_o_-F_1_ ATP synthase: a molecular motor that shapes cristae

F_o_-F_1_ ATP synthase (also referred to as Complex V), is a multimeric complex that couples the proton-motive force generated by proton pumping to ATP catalysis. The complex can be divided into two functionally distinct domains: F_1_, which contains the catalytic sites of ATP synthase; and F_o_, which is embedded in the IMM and forms a channel allowing protons to move down their electrochemical gradient [84]. The proton gradient generated by the mETC drives the rotation of a ring of c subunits in the F_o_ domain, which transmits the motion into F_1_. The rotation causes a conformational change of the alpha and beta subunits of F_1_, leading to ATP catalysis from ADP and inorganic phosphate [85]. Besides its role in energy conversion, F_o_-F_1_ ATP synthase also plays a role in controlling mitochondrial morphology. F_o_-F_1_ ATP synthase oligomerisation induces membrane curvature, facilitates cristae formation, and controls cristae shape – which likely affects the respiration rate [12,84,86,87].

In opisthokonts the F_o_-F_1_ ATP synthase enzyme consists of 18 different types of protein. The F_1_ domain is composed of five subunits, with an additional regulatory subunit that binds to the domain, and 12 proteins constitute the F_o_ portion [88,89]. Comparative genomics have shown that, in most eukaryotic lineages, the F_1_ subunits are well conserved and can be readily identified [90,91]. The function of those subunits is likely universally conserved. For example, a genetic study in *T. gondii* showed that one of those conserved subunits, the stator b subunit homologue, is essential for proper F_o_-F_1_ ATP synthase assembly, mitochondrial function, and survival of the parasite [92], as is the case in other organisms. In contrast, subunits of the F_o_ domain seem to be phylum-specific, with a poor or complete lack of subunit conservation between different organisms [93,94]. For example, in *T. gondii*, F_o_-F_1_ ATP synthase has 32 subunits, and only 15 of those have a structural equivalent found in other enzymes [9,38,41,92,95]. The remaining 17 subunits are all part of the F_o_ domain, and most of them are conserved throughout mitochondriate myzozoans [9] (Figure 3). Interestingly, a structure of the *T. gondii* F_o_-F_1_ ATP synthase showed that the enlarged F_o_ domain gives the complex unique characteristics, such as the presence of a matrix-exposed portion termed the wing region, along with an expanded lumenal region [9]. The structure revealed that myzozoan-specific subunits make up the new lumenal domain, which mediates complex hexamerisation. The resulting hexamers assemble into pentagonal pyramids that contribute to the unique, bulbous cristae morphology found in apicomplexans [9]. Since the shape of cristae is proposed to control respiration rates, these findings raise the question of how exactly this unique higher-order oligomerisation, and resulting shape, is linked to the control of respiration. Further, some of the new lumenal components contain coiled-coil-helix-coiled-coil-helix domains (CHCHD). These domains, which are implicated in numerous mitochondrial functions, including mETC biogenesis, in opisthokonts, have never been found associated with F_o_-F_1_ ATP synthase before [9,92,96]. It will be of interest to explore whether they have additional roles besides their structural functions, and to test if these domains might also contribute to the control of cristae shape and dynamics.

## New subunits, new functions?

Recent biochemical and proteomic studies have led to significant progress in our knowledge of apicomplexan respiratory chain composition. The most notable discovery is the expansion of complex size and subunit number in this group compared to the opisthokont complexes. These discoveries build a foundation for hypotheses about the evolution of the complexes in eukaryotes, and inspire hypotheses about new functions not seen in other organisms, which structural and genetic studies are beginning to address and validate. In many cases, the newly identified components are conserved beyond Apicomplexa, in other Myzozoa (Figure 3).

Several possibilities could be discussed when considering the forces that led to this expansion of respiratory complex subunit number. The first focuses on protein hydrophobicity. Myzozoan mitochondrial genomes are highly reduced, encoding only three, or even two [16], proteins. Part of the process leading to this reduction involved migration of mitochondrially encoded genes into the nuclear genome, whereby the encoded proteins are then imported back into the mitochondrion. It was proposed that within this gene transfer process the nuclear encoded proteins must become less hydrophobic to enable or enhance their mitochondrial import [97]. The split of a former mitochondrially encoded protein into several nuclear encoded proteins may supports this hypothesis. Evidence for this is seen in two examples from the apicomplexan respiratory complexes: first is the split of Cox2 into two separate proteins [77,78], and second is the truncation of F_o_-F_1_ ATP synthase subunit a, which results in its reduced hydrophobicity, and which was accompanied by the acquisition of new subunits that fill in the structural role of the missing domains [9].

A second explanation is that new subunits provide new functions that satisfy unique requirements of the organism, such as the ability to fine tune the respiration rate through controlling the shape of the cristae or the interactions between mETC complexes. *T. gondii* F_o_-F_1_ ATP synthase provides an example for this scenario. The myzozoan-specific F_o_-F_1_ ATP synthase subunits mediate the formation of its unique hexamer, which in turn forms the pentagonal pyramid that is necessary for the formation of the typical apicomplexan bulbous cristae. Why and when the bulbous shape is important for apicomplexan mitochondrial biology, and whether this is critical to control respiration rates or other aspects of IMM biology, remain to be investigated. Another example for new functions provided by newly described subunits in a noncommon model organism is provided in the recent structural study of mETC complexes in the ciliate *T. thermophila*. The Tt-CIV structure revealed many additional subunits compared to opisthokont Complex IV [2,3], some of which form a hexameric α-propeller domain composed of small TIM8-like subunits that are suggested to play a role as intermembrane space (IMS) chaperones. This addition to the *Tetrahymena* Complex IV may reflect a permanent incorporation of assembly factors into an mETC complex as a new function [2].

In other eukaryotes, mETC complexes often associate together into higher-order assemblies called supercomplexes [98,99]. These supercomplexes have been hypothesised to contribute to the efficiency of electron transport through the mETC, have a role in the stabilisation of complexes, regulate the complex’s activity, or minimise the production of reactive oxygen species [98,99]. Whether any new subunits mediate the formation of mETC supercomplexes remains an open question. In the opisthokonts, numerous supercomplex compositions have been detected, including between Complex I, dimeric Complex III, and Complex IV (also referred to as the respirasome), between Complex I and dimeric Complex III, and between dimeric Complex III and monomeric and dimeric Complex IV [51,99]. Due to the absence of Complex I in Myzozoa, many of these supercomplex compositions are not possible, suggesting that a Complex III–Complex IV supercomplex may be the dominant form (see Outstanding questions). Native page migration provided evidence for a higher molecular weight complex that includes Complex III component in *Toxoplasma* [38]. Likewise, complexome profiling performed in *P. falciparum* detected supercomplexes that consist of Complex III–Complex IV [41]. Interestingly, these supercomplexes were more abundant in the cristate gametocyte stage compared to the asexual blood stages [41]. An intriguing possibility arising from these observations is that supercomplex formation plays a role in regulating respiratory activity between the different life stages of the apicomplexan complex life cycle. Structural work will be key for mapping these interactions and deciphering the role of the new subunits.

Another possibility is that new subunits are required for complex stability, either of the individual complexes, or of the supercomplex. This is similar to the situation seen in mitoribosomes, where recent structural studies have shown numerous examples in which unicellular eukaryotes have many additional subunits compared to the mammalian version. These additional subunits have been proposed to help stabilise ribosomal RNAs and thus maintain complex integrity [100,101]. Finally, the concept of ‘new’ or ‘additional’ components is relative, as these terms are often used in comparison to the opisthokont complexes. The common ancestor of eukaryotes contained respiratory complexes that diversified through subunit losses, gains, and alterations in each of the major eukaryotic lineages. The respiratory complexes found in opisthokonts may also be highly divergent from the ancestral eukaryote. For example, the small, four-subunit Complex II of opisthokonts appears to be a streamlined version of the much larger Complexes II found in the other eukaryotic lineages. In this sense, it is the small Complex II of opisthokonts that is divergent compared to larger Complex II of other eukaryotes. It will be of interest to explore if the overall structure and function of new features of Complex II (see Outstanding questions), and indeed all respiratory complexes from these different clades, are conserved, which would indicate convergent evolution. This insight might pinpoint a functional reason for the reduced size of complexes in mammals and fungi.

## Concluding remarks

The divergent respiratory chain of apicomplexans and other myzozoans highlights the value of understanding cell biology from an evolutionary perspective [102]. Appreciating the full diversity of respiratory complex biology, and the diversity of eukaryotic biology more generally, provides insights into core biological processes in eukaryotes: how they function, how they evolve, and which features are truly universal and conserved as opposed to lineage-specific innovations, or losses.

Over the previous decades, opisthokont species have been the primary models for understanding respiratory complexes and are considered the ‘textbook’ examples of what these complexes look like and how they function. Using these model systems has undoubtedly contributed to breakthroughs in mechanistic understanding, but perhaps at the expense of a full picture of eukaryotic biology. Given the advances in genomics and genetic tools, a bigger pool of organisms can now be used as models. By harnessing these new opportunities for comparative biology, we may get a fuller picture of eukaryotic diversity and move away from the ‘textbook’-centric view of the respiratory chain.

Understanding the differences that exist in these fundamental processes can also have practical outcomes. Myzozoans include many parasites of humans and livestock that cause diseases with major global impacts. Identifying divergent features in essential processes like the mETC, and understanding these processes at a molecular level, may inform future drug discovery and development efforts against these nefarious organisms (see Outstanding questions).

## Highlights

Apicomplexan mitochondrial electron transport chain (mETC) complexes and F_o_-F_1_ ATP synthase are highly divergent compared to other commonly studied models.

Complex II, IV and F_o_-F_1_ ATP synthase in apicomplexans have significantly more subunits than their equivalents in opisthokonts (the eukaryotic group that includes yeast and humans).

The reasons for larger mETC complexes and F_o_-F_1_ ATP synthase in apicomplexans, and the functional roles of novel proteins in these complexes, remain open questions, with some recent clues starting to emerge from structural and genetic studies.

## Outstanding questions

What is the architecture of mETC supercomplexes in apicomplexans, and how do they contribute to mitochondrial biology?

Apicomplexan mETC complexes and F_o_-F_1_ ATP synthase contain additional and divergent subunits compared to their opisthokont parallels. What functions do these additional subunits serve?

Apicomplexan Complex II contains many additional subunits compared to the opisthokont complex. This is the case for organisms from other clades of the eukaryotic tree. Does the opisthokont complex represent a minimal functional core for complex II?

Can knowledge of the differences between the apicomplexan and mammalian mETC complexes be exploited in drug discovery approaches?

## Glossary

Apicomplexa: a phylum of unicellular parasites which includes pathogens responsible for numerous human and veterinary diseases; for example: *Toxoplasma gondii*, responsible for toxoplasmosis; *Plasmodium* spp., which can cause malaria; and *Cryptosporidium*, the causative agent of cryptosporidiosis.
F_o_-F_1_ ATP synthase: an F-type mitochondrial enzyme which catalyses the conversion of ADP and inorganic phosphate to ATP, using the proton gradient produced by the mETC.
Mitochondrial electron transport chain (mETC): a series of multisubunit protein complexes in the inner mitochondrial membrane which, along with the mobile electron carriers, ubiquinone and cytochrome *c*, transport electrons to oxygen, the final electron acceptor. This can be coupled to proton transport across the inner mitochondrial membrane which is utilised by ATP synthase for ATP production.
Mitochondrion: an endosymbiosis-derived organelle of eukaryotic which houses the cellular machinery for numerous important reactions, for example oxidative phosphorylation and iron-sulfur cluster synthesis.
Myzozoa: a grouping of unicellular eukaryotes, within the alveolates, which includes apicomplexa, dinoflagellates, chromerida, and perkinsozoa.
Opisthokonta: a grouping of eukaryotes which includes fungi and metazoans; it includes mammals.
Oxidative phosphorylation (OXPHOS): a series of reactions which play a key role in energy metabolism in most eukaryotic cells. Here, the mETC and F_o_-F_1_ ATP synthase are used in the synthesis of ATP, the so-called universal energy currency of the cell.
Plasmodium: a genus of apicomplexan parasites which can cause malaria in a wide range of animals. One of the key, and most deadly, species is *Plasmodium falciparum*.
Supercomplex: the association of individual mETC complexes into higher-order assemblies. An example is the respirasome, which includes Complexes I, III_2_, and IV.
*Toxoplasma gondii*: a highly prevalent obligate intracellular apicomplexan parasite of humans and most warm-blooded animals. It has become a key model organism for apicomplexan research due to its ease of culturing and range of tools for genetic manipulation.
